# Neuropsychiatric adverse events of SGLT2 inhibitors: a pharmacovigilance analysis of the FDA adverse event reporting system database

**DOI:** 10.3389/fmed.2026.1799557

**Published:** 2026-05-29

**Authors:** Minghui Yin, Hongyi Tan, Shan Xie, Yun Kuang, Qian Wu, Junlong Ma, Chengxian Guo

**Affiliations:** Center for Clinical Pharmacology, The Third Xiangya Hospital, Central South University, Changsha, Hunan, China

**Keywords:** FDA adverse event reporting system database, neuropsychiatric adverse events, pharmacovigilance analysis, risk signals, SGLT2 inhibitors

## Abstract

**Objective:**

To systematically analyze the occurrence features, temporal trends and risk signals of neuropsychiatric adverse events (AEs) linked to three sodium-glucose co-transporter 2 (SGLT2) inhibitors (canagliflozin, dapagliflozin, empagliflozin), and provide evidence for clinical safe medication.

**Methods:**

We extracted primary suspected (PS) only SGLT2 inhibitor-related reports from the FAERS database (2015Q4–2025Q3) after standardized deduplication. Descriptive analysis, dual disproportionality analysis (*ROR* and *IC*), time-stratified sensitivity analysis, and indication-based subgroup stratification were conducted.

**Results:**

A total of 141,555 eligible AE reports were included, with 11,071 neuropsychiatric AEs (7.82%). Annual trends differed; males and 45–65-year-olds were high-risk. Diabetic neuropathy was a class-wide signal; each drug had unique signals. Time-stratified analysis confirmed signal robustness. Central nervous system vascular disorders were a cross-indication core signal.

**Conclusion:**

The three SGLT2 inhibitors have non-negligible neuropsychiatric risks with population/indication differences and class/drug-specific signals. All findings represent exploratory safety signals and do not confirm causal relationships between SGLT2 inhibitors and neuropsychiatric adverse events. Limitations include no indication confounding adjustment or multiple testing correction, as well as inherent flaws of FAERS such as reporting bias, underreporting, and outcome heterogeneity. Targeted monitoring is warranted for clinical awareness, but changes in clinical practice require further confirmation in large observational cohorts or prospective studies, especially for elderly/non-diabetic patients on dapagliflozin.

## Introduction

Sodium-glucose co-transporter 2 (SGLT2) inhibitors, by inhibiting renal glucose reabsorption and promoting urinary glucose excretion, have become a crucial class of drugs for improving glycemic control in patients with type 2 diabetes mellitus (T2DM) (1). Beyond glycemic benefits, they have also demonstrated significant advantages in reducing the risk of cardiovascular and renal events, with their indications extended to heart failure (HF) and chronic kidney disease (CKD) (2, 3). Currently, dapagliflozin, empagliflozin, and canagliflozin are recommended for T2DM patients with cardiovascular or renal complications, highlighting their cross-indication therapeutic value (4). Ertuglifozine, the fourth SGLT2 inhibitor approved for clinical use in the European Union, is not included in this study due to extremely limited clinical application data and rare reports in the FAERS database (far less than the minimum sample size *n*≥3 required for disproportionality analysis), which cannot support valid pharmacovigilance signal detection.

However, with increasingly widespread clinical use, potential neuropsychiatric safety concerns associated with SGLT2 inhibitors have gradually garnered attention. Multiple case reports suggest that some patients may experience neuropsychiatric symptoms such as dizziness, headache, somnolence, anxiety, and depression after medication use (5) Mechanistically, it is speculated that these drugs may affect the nervous system through pathways such as interfering with neurotransmitter metabolism (e.g., affecting the balance of gamma-aminobutyric acid and glutamate), altering blood-brain barrier function, or impacting central energy metabolism (6, 7). However, due to the non-specific nature of these symptoms, they are often confused with complications of diabetes itself or symptoms of other comorbidities, making clinical identification difficult and potentially leading to an underestimation of the associated risk (8, 9).

Current research on SGLT2 inhibitors primarily focuses on cardiovascular and renal outcomes, with neuropsychiatric safety data largely stemming from small-scale studies or scattered case reports (10). In contrast, the neuropsychiatric risks of another class of glucose-lowering drugs, glucagon-like peptide-1 receptor agonists (GLP-1RAs), have been relatively well-investigated (11, 12). Therefore, conducting a systematic pharmacovigilance analysis specifically for SGLT2 inhibitors is particularly necessary.

The US Food and Drug Administration Adverse Event Reporting System (FAERS), as one of the largest global spontaneous reporting databases for adverse drug reactions, provides a vital platform for real-world drug safety research (13). This study aims to systematically evaluate the occurrence characteristics, risk signals, and temporal patterns of neuropsychiatric adverse reactions associated with SGLT2 inhibitors by mining and analyzing data from the FAERS database spanning 2015Q4 to 2025Q3, thereby providing evidence-based guidance for clinical safe medication use.

## Methods

### Data source

We conducted a pharmacovigilance study on neuropsychiatric adverse events (AEs) associated with sodium-glucose cotransporter 2 inhibitors (SGLT-2I) based on the FAERS database, a publicly available database of safety reports submitted by patients, healthcare professionals, and pharmaceutical companies (14). Only cases with SGLT-2I as the “primary suspected (PS)” drug were included in this study, and cases with SGLT-2I as the secondary suspected (SS) drug were strictly excluded. The inclusion of only PS reports was based on three core scientific rationales: first, the primary objective of this study was to identify neuropsychiatric AEs with a potential causal association with SGLT2 inhibitors, and PS reports represent the reporter's clinical judgment that the drug is the primary cause of the AE, which is the gold standard for causal association analysis in spontaneous reporting database (SRD) pharmacovigilance studies (14, 15); second, the inclusion of SS reports would introduce severe confounding bias, as SS drugs may be the actual causal agent of neuropsychiatric AEs and lead to misclassification of SGLT2 inhibitor-associated risks; third, this inclusion criterion is consistent with the majority of high-quality pharmacovigilance studies on SGLT2 inhibitors and glucagon-like peptide-1 receptor agonists (GLP-1RAs) (11, 16), ensuring the comparability of our results with existing literature. We downloaded the FAERS data files from 2015Q4 to 2025Q3 and identified SGLT-2I drugs by their generic and brand names, including Dapagliflozin (Dapagliflozin Viatris, Ebymect, Edistride, Farxiga, Forxiga, Qtern, Qternmet, Xigduo), Empagliflozin (Glyxambi, Jardiance, Synjardy, Trijardy), and Canagliflozin (Invokamet, Invokana).

Additionally, all adverse reactions recorded in the FAERS database were coded using the Medical Dictionary for Regulatory Activities (MedDRA) Preferred Term (PT) coding system (17). This dictionary is divided into five distinct hierarchical levels, where Preferred Terms (PTs) serve as unique identifiers for specific medical concepts, such as symptoms, signs, and disease diagnoses. In addition to PTs, the hierarchical system further classifies medical concepts by incorporating “High-Level Terms (HLTs)” and “High-Level Group Terms (HLGTs)”. Ultimately, these HLGTs are categorized into “System Organ Classes (SOCs)” based on their origin, site of presentation, or purpose. Due to its multiaxial structure, different PTs can be classified into discrete SOCs, with a primary SOC assigned to each classification.

Based on this framework, we focused our analysis on neuropsychiatric PTs associated with “psychiatric disorders” and “nervous system disorders” as the primary System Organ Classes (SOCs). Specifically, we extracted PTs corresponding to all psychiatric AEs (*N* = 590) and nervous AEs (*N* = 1,120) from MedDRA version 27.1.

### Data processing procedure

We performed deduplication of the SGLT-2I-related PS reports obtained from the FAERS database using a standardized stepwise algorithm recommended by Banda et al. (15), with adaptations to resolve discrepancies in case identifiers, follow-up versions, and missing data; the detailed deduplication steps were as follows: (1) Extraction of core matching variables: six core variables with high specificity for identifying duplicate clinical cases were extracted for all reports, including case ID, case initial/follow-up code (“I” for initial version, “F” for follow-up version), case event date, patient age, patient sex, and reporting country. (2) Resolution of case identifier discrepancies: for reports with duplicate case IDs but inconsistent clinical information (≈1.2%), the report with a complete AE onset date was prioritized; if all onset dates were complete, the report with the most detailed AE description was retained. Reports with missing case IDs (≈0.8%) were excluded due to unreliable matching. (3) Prioritization of follow-up versions: for reports with the same case ID and consistent clinical variables but different version codes, the latest follow-up version (F) was retained and the initial version (I) was excluded; for reports with multiple F versions (≈0.5%), the version with the most recent submission date (extracted from the FAERS data file header) was retained. (4) Handling missing data in matching variables: for reports with partial missing non-critical variables (e.g., reporting country), the report with the most complete variable set was retained; for reports with missing key variables (AE onset date + case version code, ≈2.1%), they were excluded from subsequent analysis due to the inability to perform time-to-onset calculation and version prioritization. (5) Final duplicate removal: exact matching of the six core variables was performed after resolving discrepancies and missing data, and only the most complete and up-to-date version of each unique case was retained for the final analysis. A total of 28,763 duplicate reports (≈16.8% of the initial raw data) were identified and removed via this algorithm.

### Descriptive analysis and time-to-onset analysis

We performed a descriptive analysis of the clinical characteristics of the screened SGLT-2I-related neuropsychiatric AE reports, including sex, age, age group, reporter type, report year, indication, outcome, and time-to-onset groups. Time-to-onset was defined as the interval between the initiation of SGLT-2I administration and the occurrence of an AE. When calculating time-to-onset, we only selected data with an onset time greater than 0 days; reports with incorrect dates (i.e., dosing time later than the event time) and missing dates were excluded.

### Disproportionality analysis

In pharmacovigilance studies, disproportionality analysis is primarily used to evaluate the potential association between a specific AE and a particular drug. we conducted 2 dimensionality (2D) disproportionality analyses using both the reporting odds ratio (*ROR*) and information component (*IC*) methods to identify safety signals for each SGLT2 Inhibitors. When reports involved multiple drugs and/or multiple AEs, we used the report unit instead of the drug-event combination. To reduce false negative signals, we employed statistical shrinkage transformation. The signal was recognized when the number of AEs was at least 3. A positive signal was identified when both the lower limit of the 95% confidence interval for *ROR* (ROR025) was greater than 1 and the lower limit of the 95% confidence interval for *IC* (IC025) exceeded 0. This threshold defines a positive pharmacovigilance signal rather than formal statistical significance (Tables 1, 2).

**Table 1:** Formulas for Disproportionality Analysis using ROR and IC methods.

Method	Formula
*ROR*	*ROR* = $\frac{a/b}{c/d}$
*IC*	*IC* = ${\text{log}}_{2}\frac{a\left(a+b+c+d\right)}{\left(a+b\right)\left(a+c\right)}$

**Table 2:** 2 × 2 Contingency Table for Disproportionality Analysis.

Category	SGLT-2I	All other drugs in the database
Neuropsychiatric adverse events	a	c
All other adverse events in the database	b	d

where a is the number of neuropsychiatric AEs cases involving SGLT-2I;

b is the number of all other AE case reports involving SGLT-2I;

c is the number of neuropsychiatric AE cases involving all other drugs in the whole database;

d is the number of other AE cases involving all other drugs in the whole database.

In the disproportionality analysis, we first conducted a comprehensive assessment of SGLT-2I-related neuropsychiatric AEs based on three pre-defined indication subgroups [diabetes mellitus (DM), cardiovascular diseases (CVD), chronic kidney disease (CKD)], detailed in Supplementary Table S1. The subgroup classification was strictly based on FAERS free-text notes and MedDRA Preferred Terms (PTs), with only cases with clear records of primary clinical indication included; cases with missing or ambiguous indication information were excluded. Stratified *ROR* and *IC* calculation was adopted for each subgroup. Subsequently, according to the MedDRA dictionary, we selected nine important neuropsychiatric signals including Central nervous system vascular disorders, Cranial nerve disorders (excl neoplasms), Disturbances in thinking and perception, Headaches, Mental impairment disorders, Neurological disorders NEC, Neuromuscular disorders, Schizophrenia and other psychotic disorders and Suicidal and self-injurious behaviors NEC and aggregated the relevant AE groups at the High-Level Group Term (HLGT) level. This approach ensured that the analyzed PTs were clinically relevant and accurately reflected actual psychiatric and nervous AEs, thereby enhancing the validity of the subsequent analysis.

To further validate the robustness of neuropsychiatric adverse event risk signals and exclude the interference of temporal changes in overall FAERS reporting volume, a time-stratified sensitivity analysis was performed at the MedDRA High-Level Term (HLT) level. The entire study period (2015Q4 to 2025Q3) was divided into six consecutive time intervals: 2015Q4–2016, 2017–2018, 2019–2020, 2021–2022, 2023–2024, and 2025Q1–2025Q3. Disproportionality analysis using the Reporting Odds Ratio (*ROR*) and Information Component (*IC*) was conducted separately for each time stratum, with the same positive signal judgment criteria as the main analysis. This stratification was applied to adjust for the dynamic changes in overall adverse event reporting volume over time, and to clarify whether the observed annual increasing trend of neuropsychiatric AEs was attributed to genuine drug-related risk signal amplification rather than global reporting dynamics.

## Results

### Descriptive analysis

**Figure 1:**
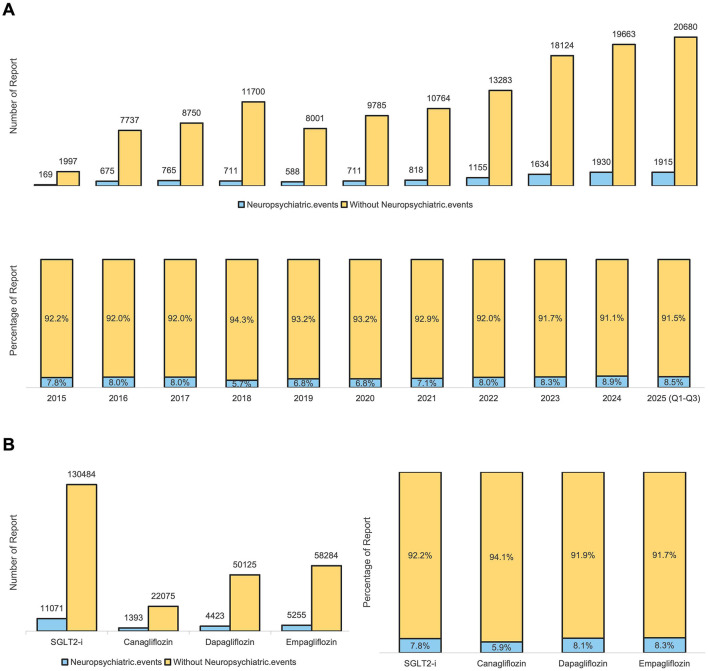
Data on reported cases of Sodium-glucose cotransporter two inhibitors (SGLT-2i)-related neuropsychiatric adverse events (AEs) from the FDA Adverse Event Reporting System (FAERS) database between 2015 and 2025. **(A)** The lower bar plot depicts the number of SGLT-2i reports with neuropsychiatric AEs vs. those without for each year in the FAERS database from 2015Q4 to 2025Q3. The proportional bar chart below illustrates the ratio of SGLT-2i reports with neuropsychiatric AEs compared to those without for each year in the FAERS database during 2015Q4 to 2025Q3. Blue denotes reports with neuropsychiatric AEs, while yellow denotes those without. **(B)** The lower bar plot presents the number of SGLT-2i reports involving neuropsychiatric adverse reactions vs. those without for various SGLT-2i treatment strategies in the FAERS database from 2015Q4 to 2025Q3. The proportional bar chart beside shows the proportion of SGLT-2i reports with neuropsychiatric AEs compared to those without for different SGLT-2i in the FAERS database from 2015Q4 to 2025Q3.

Figure 1 show that from 2015Q4 to 2025Q3, the total number of adverse events (AEs) of the three SGLT2 inhibitors (canagliflozin, dapagliflozin, and empagliflozin) accumulated to 141,555; among them, the cumulative number of neuropsychiatric adverse reactions was 11,071, accounting for 7.82% of the total AEs. As presented in Table 3, there were significant differences in the annual trends of psychiatric AEs and their proportions relative to the total AEs of each drug in the same year among the three drugs: dapagliflozin showed a trend of continuous and substantial growth, increasing year by year from 53 cases in 2015 (accounting for 7.92%) to 1,136 cases in 2025 (accounting for 8.54%), with the most significant growth rate and a slight overall upward trend in the annual proportion; empagliflozin showed a trend of steady increase followed by a slight decline, with 30 cases in 2015 (accounting for 9.23%), peaking at 877 cases in 2024 (accounting for 8.24%), and slightly decreasing to 741 cases in 2025 (accounting for 8.45%).

**Table 3:** Characteristics of reports with SGLT-2i related neuropsychiatric adverse events sourced from the FDA adverse event reporting system database (2015Q4 to 2025Q3).

Characteristic	SGLT-2i	Canagliflozin	Dapagliflozin	Empagliflozin
Sex				
F	4,512	566	1,888	2,058
M	5,480	680	2,135	2,665
Missing	1,082	148	400	534
Age				
≥75	1,694	113	872	709
65–75	1,789	189	602	998
45–65	2,690	469	901	1,320
18–45	574	88	202	284
<18	90	32	15	43
Missing	4,237	503	1,831	1,903
Outcome				
OT	3,718	414	1,769	1,535
HO	2,589	393	1,022	1,174
LT	623	42	307	274
DE	381	44	162	175
DS	279	30	134	115
RI	9	1	1	7
CA	3	0	2	1
Missing	3,472	470	1,026	1,976
Report year				
2015Q4	169	86	53	30
2016	675	319	173	183
2017	765	321	176	268
2018	711	186	187	338
2019	588	113	139	336
2020	711	100	194	417
2021	818	56	255	507
2022	1,155	53	409	693
2023	1,634	72	697	865
2024	1,930	49	1,004	877
2025 (Q1–Q3)	1,915	38	1,136	741
The time to onset (days)				
>360	734	179	211	344
181–360	405	134	130	141
151–180	131	31	45	55
121–150	118	24	47	47
91–120	148	36	53	59
61–90	212	60	75	77
31–60	350	87	118	145
0–30	2,475	572	818	1,085
<0	926	247	491	188
Unknown or missing	7,724	1,351	3,106	3,267
Diabetes mellitus or blood glucose abnormal	15,364	4,346	5,648	5,370
Others	20,906	1,357	14,639	4,910
Unknown or missing	4,713	196	3,692	825

F, female; M, male; OT, other outcomes; HO, hospitalization; LT, life-threatening; DE, death; DS, disability; RI, requirement for intervention; CA, carcinogenesis.

Table 3 also presents the distribution characteristics with proportional differences of neuropsychiatric adverse events associated with three SGLT2 inhibitors (canagliflozin, dapagliflozin, and empagliflozin) across four dimensions: sex, age, outcome, and indication. In terms of sex, male reports accounted for a higher proportion for all three drugs, with the male proportions of canagliflozin, dapagliflozin and empagliflozin standing at 54.6, 53.1 and 56.4% respectively, and the female proportions at 45.4, 46.9 and 43.6%. For age distribution, the 45–65 age group was the high-incidence population for such adverse events among the three drugs, accounting for 33.7, 20.4 and 25.1% respectively. Dapagliflozin had a notably higher proportion of reports in the elderly population aged ≥75 at 19.7%, compared with 8.1% for canagliflozin and 13.5% for empagliflozin, while the proportion of reports in the adolescent population under 18 was less than 5% for all three drugs. Regarding outcomes, non-serious outcomes dominated for all three drugs, mainly including other outcomes (OT) and hospitalization (HO): the proportions of other outcomes for canagliflozin, dapagliflozin and empagliflozin were 30.0, 40.0 and 29.2% respectively, and the proportions of hospitalization were 28.2, 23.1 and 22.3% respectively. Severe outcomes such as death and disability accounted for less than 5% of the reports for each drug, and extremely rare cases of outcomes like carcinogenesis and birth defects were only seen in individual drugs. In terms of indication, diabetes mellitus or abnormal blood glucose was the primary clinical background for the use of all three drugs, corresponding to proportions of 93.4, 27.5 and 83.5% respectively. Notably, dapagliflozin had a markedly higher proportion of reports in other indications at 71.0%, far exceeding that of canagliflozin and empagliflozin. Categorized into nine intervals by days of onset, the 0–30 days post-administration period was the peak interval for adverse events across all three drugs, with the 31–60 days interval being the second most frequent, and empagliflozin recording the highest number of reports among the three drugs in both intervals. Overall, the number of reports showed a gradual decrease as the onset interval extended, yet a large number of reports had unknown or missing onset time.

### Disproportionality analysis

Based on the disproportionality analysis in this study, adverse event signals associated with SGLT2 inhibitors exhibit distinct patterns of class-wide commonality, drug specificity, and contradictory signals.

**Figure 2:**
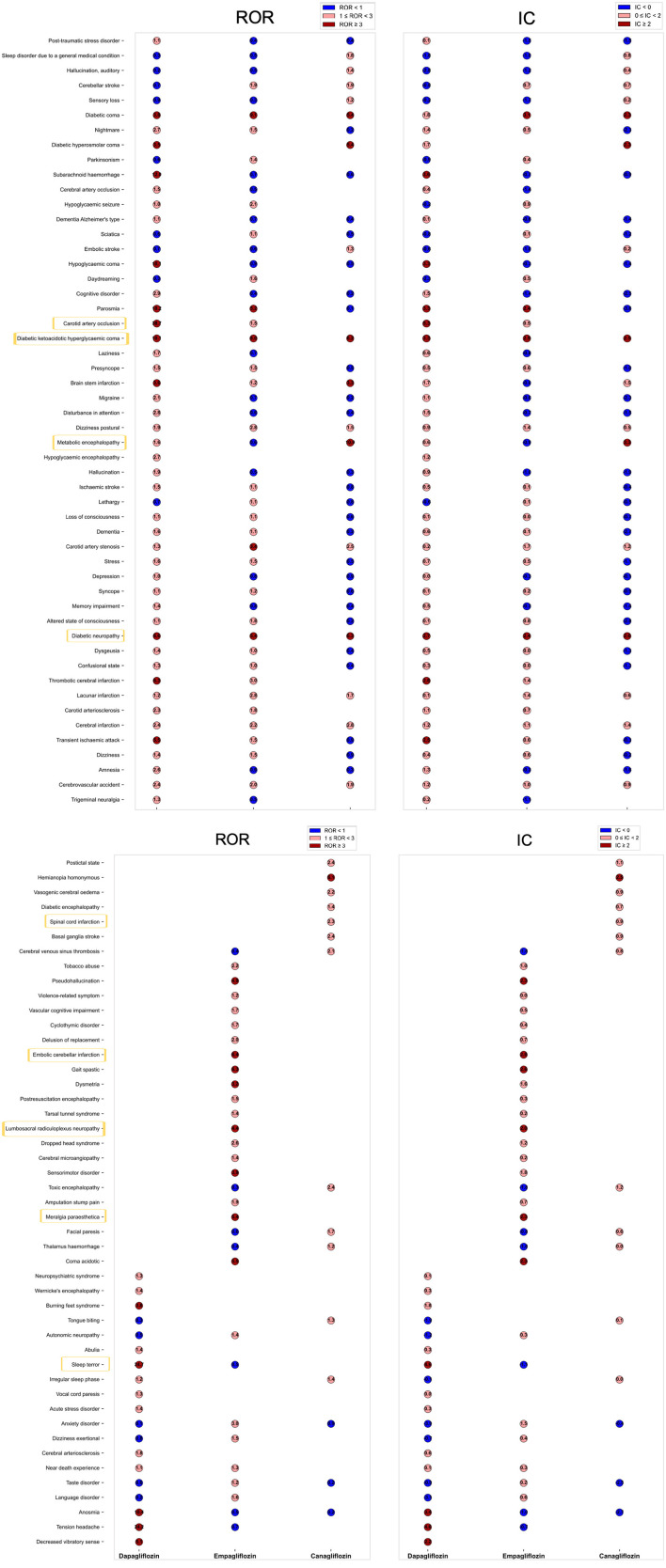
In the FAERS database under different SGLT-2i, dark red indicates reporting odds ratio (*ROR*) values greater than 3, and light red indicates *ROR* values less than 3 and greater than 1; dark blue indicates *ROR* values less than 1; white indicates *ROR* values that could not be calculated. In the FAERS database under different SGLT-2i, dark red indicates information component (*IC*) values greater than 2, and light red indicates *IC* values less than 2 and greater than 0; dark blue indicates *IC* values less than 0; white indicates *IC* values that could not be calculated. Neuropsychiatric AEs labeled with red or blue color meet the criteria that the number of cases occurring no less than 3.

The core findings revealed consistent and robust positive signals across all three agents (Figure 2). Notably, diabetic neuropathy emerged as a common key positive signal, meeting the strict positive signal criteria for all three drugs: dapagliflozin (*ROR* = 6.62, *IC* = 2.69), empagliflozin (*ROR* = 5.35, *IC* = 2.37), and canagliflozin (*ROR* = 6.33, *IC* = 2.59). All values were far above the positive threshold, indicating a clear potential association between this adverse event and SGLT-2 inhibitors as a class, marking it as a priority safety event for the entire drug class.

Analysis of agent-specific positive signals showed that dapagliflozin was associated with multiple high-intensity independent positive signals, among which several adverse events exhibited exceptionally strong signal strength: carotid artery occlusion (*ROR* = 38.69, *IC* = 5.27), diabetic ketoacidotic hyperglycaemic coma (*ROR* = 10.11, *IC* = 3.28), and sleep terror (*ROR* = 24.70, *IC* = 4.62). All these events had *ROR* and *IC* values well exceeding the positive signal threshold, with extremely high signal intensity, suggesting a particularly significant risk association between dapagliflozin and these severe neuropsychiatric and cerebrovascular adverse events, which holds core clinical pharmacovigilance value.

Empagliflozin presented several unique positive warning signals, with representative high-risk events including meralgia paraesthetica (*ROR* = 5.44, *IC* = 2.32), lumbosacral radiculoplexus neuropathy (*ROR* = 4.44, *IC* = 2.01), and embolic cerebellar infarction (*ROR* = 6.42, *IC* = 2.81). All indicators strictly conformed to the positive signal criteria, forming distinct safety characteristics of empagliflozin that differentiate it from the other two agents, and providing key data support for individualized clinical risk prevention and control of this drug.

Canagliflozin also exhibited specific positive association signals, with homonymous hemianopia (*ROR* = 6.14, *IC* = 2.48) and metabolic encephalopathy (*ROR* = 9.97, *IC* = 3.26) showing the most significant signal strength, and spinal cord infarction (*ROR* = 2.30, *IC* = 0.92) also meeting the positive signal criteria. These findings indicate that such severe neurological adverse events are specific risk priorities requiring close monitoring for canagliflozin, and can offer targeted safety references for precise clinical medication.

### Description of results of time-stratified sensitivity analysis at HLT level

**Figure 3:**
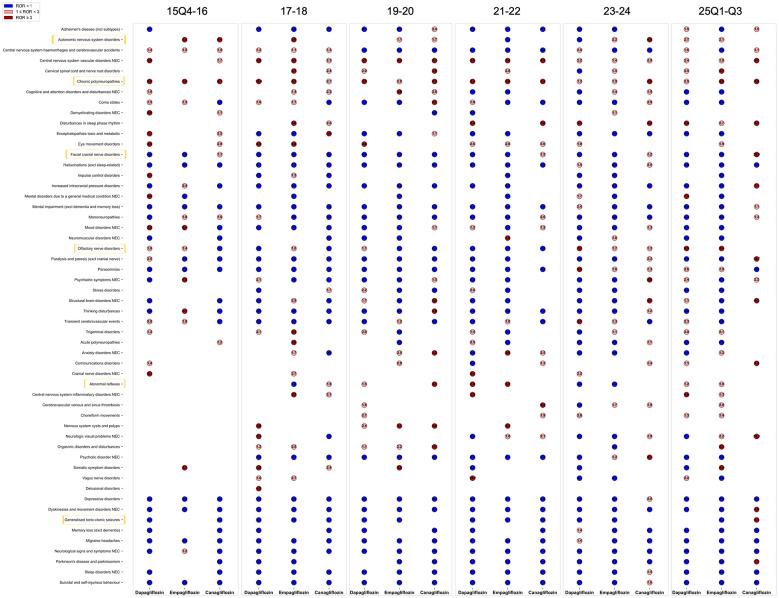
Time-stratified sensitivity analysis results of neuropsychiatric adverse event risk signals at HLT level for three SGLT2 inhibitors. The study period (2015Q4–2025Q3) was divided into six time periods. The figure shows the met the positive signal criteria (ROR025>1) of 54 neuropsychiatric system-related HLTs for empagliflozin, dapagliflozin, and canagliflozin in most time periods, confirming the robustness of class-wide common signals (e.g., chronic polyneuropathies) and drug-specific risk signals. Colors as in Figure 2.

**Figure 4:**
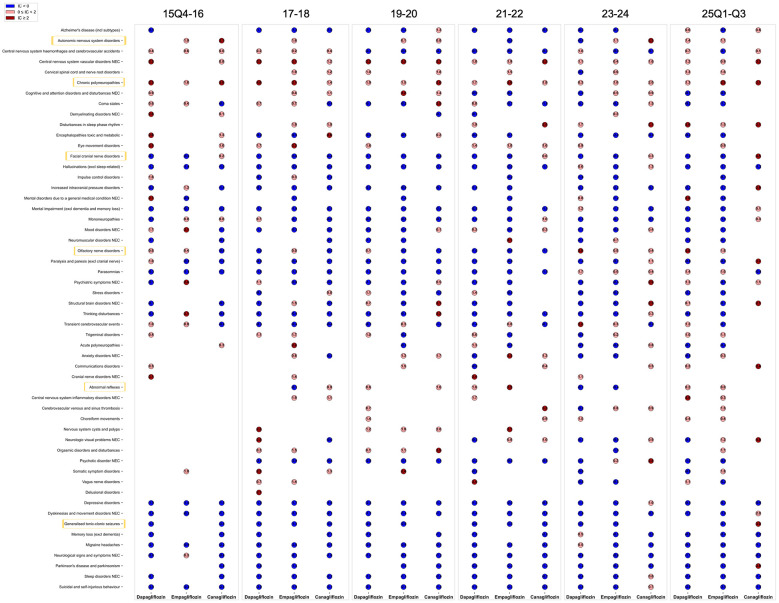
Time-stratified sensitivity analysis results of neuropsychiatric adverse event risk signals at HLT level for three SGLT2 inhibitors. The study period (2015Q4–2025Q3) was divided into six time periods. The figure shows the met the positive signal criteria (IC025>0) of 54 neuropsychiatric system-related HLTs for empagliflozin, dapagliflozin, and canagliflozin in most time periods, confirming the robustness of class-wide common signals (e.g., chronic polyneuropathies) and drug-specific risk signals. Colors as in Figure 2.

Figures 3, 4 shows the results of the time-stratified sensitivity analysis based on MedDRA High-Level Terms (HLT), focusing on HLTs mentioned in the main text (chronic polyneuropathies, olfactory nerve disorders, abnormal reflexes, etc.). The analysis divided the period from 2015Q4 to 2025Q3 into six time periods, and analyzed the *ROR* and *IC* values of key neuropsychiatric system-related HLTs for three drugs, focusing on describing signals that met the positive signal criteria (ROR025>1 and IC025>0) in most time periods. Among them, chronic polyneuropathies was a stable positive signal common to all three drugs, with positive results in all six time periods, and the signal intensity was particularly prominent in some time periods; in addition, HLTs such as olfactory nerve disorders and abnormal reflexes showed positive results in five or four time periods for all three drugs, collectively forming the core risk signals of neuropsychiatric system adverse reactions for this class of drugs. Each single drug also had prominent specific signals: autonomic nervous system disorders of Empagliflozin were positive in multiple time periods, and psychiatric symptoms were strongly positive only in a single time period; demyelinating disorders of Dapagliflozin were strongly positive in a single time period, and HLTs related to mental disorders showed significant positivity in multiple time periods; HLTs such as facial cranial nerve disorders and generalized tonic-clonic seizures of Canagliflozin were strongly positive in the later time periods, and the signal intensity of some HLTs was significantly higher than that of the other two drugs. Overall, these results verified the robustness of the risk signals of neuropsychiatric system adverse reactions, providing a reference for clinical medication safety.

### Neuropsychiatric AE profiles based on indication

**Figure 5:**
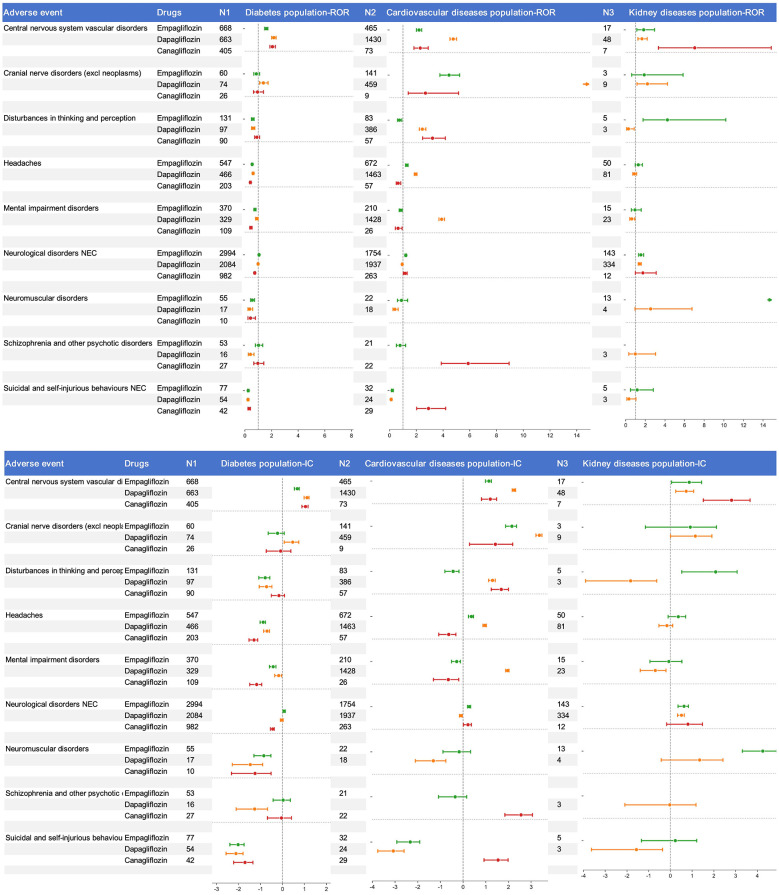
Forest plot showing the reporting odds ratio (*ROR*) and information component (*IC*) of eight specific neuropsychiatric adverse events with different SGLT-2i based on diabetes indication population, cardiovascular diseases population and kidney diseases population. N1, ROR1, IC1, Lower1, and Upper1 are data related to the diabetes population, whereas N2, ROR2, IC2, Lower2, and Upper2 are data related to the cardiovascular diseases population, and N3, ROR3, IC3, Lower3, and Upper3 are data related to the kidney diseases population.

Figure 5 illustrates the results of signal detection for neuropsychiatric adverse events classified by the MedDRA HLGT level, related to dapagliflozin, empagliflozin, and canagliflozin, across three populations: patients with diabetes, cardiovascular diseases, and kidney diseases. Central nervous system vascular disorders showed consistent and robust positive signals for all three agents in both the diabetes and cardiovascular diseases populations, fully meeting the pre-defined criteria. In the diabetes population: empagliflozin (*ROR* = 1.59, 1.48–1.72; *IC* = 0.67, 0.54–0.76), dapagliflozin (*ROR* = 2.16, 2.00–2.33; *IC* = 1.11, 0.98–1.21), and canagliflozin (*ROR* = 2.06, 1.87–2.28; *IC* = 1.05, 0.88–1.17). In the cardiovascular diseases population: empagliflozin (*ROR* = 2.20, 2.01–2.41; *IC* = 1.14, 0.98–1.25), dapagliflozin (*ROR* = 4.76, 4.51–5.01; *IC* = 2.25, 2.16–2.31), and canagliflozin (*ROR* = 2.29, 1.82–2.89; *IC* = 1.20, 0.81–1.48). This category represents a core class-wide safety signal for SGLT-2 inhibitors.

Empagliflozin exhibited population-specific high-strength positive signals, most prominently for Neuromuscular disorders in the kidney diseases population (*N* = 13, *ROR* = 19.05, 11.03–32.88; *IC* = 4.25, 3.31–4.89). Additionally, positive signals were detected for Cranial nerve disorders (excl neoplasms) in the cardiovascular diseases population (*N* = 141, *ROR* = 4.44, 3.76–5.24; *IC* = 2.15, 1.87–2.35) and Disturbances in thinking and perception in the kidney diseases population (*N* = 5, *ROR* = 4.24, 1.77–10.21; *IC* = 2.09, 0.52–3.07). No positive signals were identified in the diabetes population.

Dapagliflozin had the widest range of positive signals, mainly concentrated in the cardiovascular diseases population, including Cranial nerve disorders (excl neoplasms; *N* = 459, *ROR* = 10.29, 9.38–11.28; *IC* = 3.36, 3.21–3.47), Mental impairment disorders (*N* = 1428, *ROR* = 3.89, 3.69–4.10; *IC* = 1.96, 1.87–2.02), and Disturbances in thinking and perception (*N* = 386, *ROR* = 2.46, 2.22–2.71; *IC* = 1.30, 1.13–1.42). In the diabetes population, Cranial nerve disorders (excl neoplasms) also met the positive signal criteria (*N* = 74, *ROR* = 1.38, 1.10–1.73; *IC* = 0.47, 0.08–0.74), while no positive signals were found in the kidney diseases population.

Canagliflozin showed specific positive signals in the cardiovascular diseases and kidney diseases populations. In the kidney diseases population, Central nervous system vascular disorders had an extremely high signal strength (*N* = 7, *ROR* = 7.04, 3.34–14.84; *IC* = 2.81, 1.51–3.67). In the cardiovascular diseases population, positive signals included Schizophrenia and other psychotic disorders (*N* = 22, *ROR* = 5.88, 3.87–8.94; *IC* = 2.56, 1.84–3.06), Disturbances in thinking and perception (*N* = 57, *ROR* = 3.21, 2.47–4.17; *IC* = 1.68, 1.24–2.00), and Suicidal and self-injurious behaviors NEC (*N* = 29, *ROR* = 2.91, 2.02–4.19; *IC* = 1.54, 0.92–1.98). No positive signals were detected in the diabetes population.

## Discussion

This study mined adverse event data of three SGLT2 inhibitors (canagliflozin, dapagliflozin, and empagliflozin) from the FAERS database between 2015Q4 to 2025Q3, systematically analyzing the occurrence characteristics, risk signals, and indication-related distribution differences of their neuropsychiatric adverse events. Based on descriptive analysis and dual disproportionality analysis using the Reporting Odds Ratio (*ROR*) and Information Component (*IC*) methods, this study comprehensively clarified the class-wide common safety features and drug-specific risk profiles of neuropsychiatric adverse events associated with the three agents, filling the gap in systematic description and risk stratification in the research on neuropsychiatric safety of this class of drugs, providing important real-world evidence-based support for clinical safe medication (16).

Descriptive analysis showed that the three SGLT2 inhibitors had a total of 141,555 reported adverse events, with neuropsychiatric adverse events accounting for 7.82% (11,071 cases), indicating that they are an unavoidable safety hazard in clinical application (18). There were significant differences in the annual trends of neuropsychiatric adverse events among the three drugs: dapagliflozin increased continuously from 53 cases in 2015 to 1,136 cases in 2025 with a slight upward annual proportion, which was related to the expansion of clinical application and increased monitoring attention; empagliflozin increased steadily to a peak in 2024 and then decreased slightly, possibly due to the accumulation of long-term safety data and optimization of medication standards. The annual proportions of both remained between 7.92 and 9.23%, requiring long-term monitoring (19).

Demographic and clinical characteristics showed that the proportion of male reports was slightly higher for neuropsychiatric adverse events of all three drugs, consistent with previous research conclusions on the impact of gender differences on drug adverse reactions (20, 21), which is speculated to be related to the severity of underlying diseases and differences in drug metabolism in males. The 45–65 age group was the high-incidence population, and dapagliflozin had a significantly higher report proportion (19.7%) in the ≥75-year-old population than the other two drugs, suggesting that special attention should be paid to such adverse reactions when dapagliflozin is used in elderly patients (22). Outcomes were mainly non-severe, accounting for less than 5% of severe outcomes; in terms of indications, diabetes mellitus or abnormal blood glucose was the main medication background, and dapagliflozin had a much higher report proportion (71.0%) in other indications than similar drugs (23), requiring neuropsychiatric risk assessment when used in populations with non-diabetic indications.

Disproportionality analysis revealed three patterns of risk signals for neuropsychiatric adverse events of SGLT2 inhibitors: class-wide commonalities, drug specificity, and contradictions (24). The above findings further confirm that distinct neuropsychiatric safety profiles exist among different SGLT2 inhibitors. The consistent detection of diabetic neuropathy as a common positive signal suggests a potential class-wide effect related to the pharmacological mechanism of this category. (7, 25, 26). Meanwhile, the unique high-strength signals identified for each agent highlight the heterogeneity in adverse event risks, which may be attributed to differences in chemical structure, target selectivity, and off-target effects. These observations provide important evidence for optimizing clinical medication selection and strengthening individualized safety monitoring, helping clinicians balance therapeutic benefits with potential risks in practical application (27).

Time-stratified sensitivity analysis further verified the robustness of the above risk signals. This analysis divided the study period into six time periods, focusing on HLTs met the positive signal criteria in most time periods. Chronic polyneuropathies, as a common positive signal shared by all three drugs, showed stable positive results in all time periods; olfactory nerve disorders and abnormal reflexes also presented consistent positive signals in most time periods, forming core risk signals of neuropsychiatric adverse events. In terms of drug-specific performance, empagliflozin showed stable positive signals in autonomic nervous system disorders across multiple time periods; dapagliflozin maintained positive signals in central nervous system vascular-related HLTs in all time periods; canagliflozin had prominent positive signals in sleep phase-related HLTs, with continuous and stable signal intensity. These results confirmed that the detected neuropsychiatric risk signals were not affected by time factors, further supporting the reliability of the disproportionality analysis results.

Analysis of indication-specific neuropsychiatric adverse event profiles based on *ROR* and *IC* dual verification further clarified the population-specific risk characteristics: central nervous system vascular disorders were a core common valid risk signal for the three drugs, showing robust positive signals in both diabetic and cardiovascular disease populations, and all signals were confirmed to be valid by *IC* method, which was related to differences in the pharmacological effects of drugs in populations with different indications (28). Specifically, empagliflozin showed a super-high-strength population-specific positive signal for neuromuscular disorders in the kidney disease population (*N* = 13, *ROR* = 19.05, *IC* = 4.25), which was stably verified by both analytical methods; dapagliflozin had the widest distribution of valid positive signals, mainly concentrated in the cardiovascular disease population, with all signals meeting the dual criteria of *ROR* and *IC*; canagliflozin showed prominent specific positive signals in cardiovascular and kidney disease populations, especially the ultra-high-strength signal for central nervous system vascular disorders in the kidney disease population (*N* = 7, *ROR* = 7.04, *IC* = 2.81), with reliable verification results, providing targeted directions for medication monitoring in populations with different indications (29).

This study has important clinical significance: it can provide guidance for individualized medication and formulate differentiated monitoring plans for different populations; it reminds clinicians to strengthen medication education and improve patients' ability to identify and report symptoms; it points out targeted directions for post-marketing drug safety monitoring, focusing on the long-term neuropsychiatric safety of dapagliflozin in elderly and non-diabetic indication populations, as well as the common risk of central nervous system vascular disorders shared by the three SGLT2 inhibitors.

This study has limitations: first, only PS reports were included in this study, and SS reports were excluded, which may attenuate the signal strength of mild or low-frequency neuropsychiatric AEs and slightly underrepresent cases with multiple comorbidities and polypharmacy; however, this impact is negligible for strong drug-specific signals, and the overall distribution trend of key subgroups remains valid due to the sufficient sample size of PS reports. Second, spontaneous reports based on the FAERS database have defects such as reporting bias and incomplete data, and cannot clarify the causal relationship between drugs and neuropsychiatric adverse events (14). Third, due to the lack of complete standardized clinical covariates, detailed disease severity data and corresponding statistical parameters (including *P*-values) required for relevant adjustment, this study did not perform statistical adjustments to account for potential confounding by indication when comparing *ROR* values across diabetes, cardiovascular diseases and kidney disease populations. This may lead to potential residual confounding in the subgroup comparison results, and the relevant risk signal differences among different indication subgroups need to be interpreted cautiously. Fourth, this study did not conduct formal multiple testing correction when performing disproportionality analysis across numerous MedDRA Preferred Terms (PTs), High Level Terms (HLTs), and High Level Group Terms (HLGTs), which may increase the risk of false-positive signals. Although we adopted strict dual validation criteria using *ROR* and *IC* methods to reduce false positive results, the potential risk of inflated false-positive signals due to unadjusted multiple comparisons cannot be completely ruled out, and the detected positive signals need to be interpreted as exploratory results rather than confirmatory causal conclusions. Future studies need to verify this association through prospective cohort studies and randomized controlled trials, explore risk factors and mechanisms, and optimize individualized medication regimens combined with pharmacokinetic and genomic studies (30, 31).

In summary, all three SGLT2 inhibitors have varying degrees of risk of neuropsychiatric adverse events, with clear annual trends, and differences in population and clinical characteristics; their risk signals have both class-wide commonalities and drug specificity. Notably, all identified neuropsychiatric events represent exploratory safety signals instead of confirmed causal risks attributed to SGLT2 inhibitors, due to the inherent limitations of the FAERS database including reporting bias, underreporting, confounding by indication, and heterogeneity of neuropsychiatric terms. Clinically, monitoring and intervention should be strengthened in combination with drug characteristics, individual patient conditions and indications. However, the present findings require further validation in large-scale observational cohorts or prospective controlled trials before any modification of clinical practice. Further studies should be carried out to improve safety data, providing more sufficient evidence-based support for clinical safe and rational medication.

## Data Availability

The original contributions presented in the study are included in the article/Supplementary material, further inquiries can be directed to the corresponding author.
