# Supplementary material for: Neuropsychiatric adverse events of SGLT2 inhibitors: a pharmacovigilance analysis of the FDA adverse event reporting system database
Source: Front Med (Lausanne). 2026 May 29;13:1799557. doi: 10.3389/fmed.2026.1799557 (PMC13260334; doi:10.3389/fmed.2026.1799557)
Supplement: Supplementary file 1 [file Table_1.docx]

**Supplementary Table 1：**List of Indications Classified by Disease Category. This table summarizes the included indications categorized by each disease area.

| **Indication Subgroups** | **Indication** |
| --- | --- |
| Diabetes | Type 2 diabetes mellitus |
| Diabetes | Diabetes mellitus |
| Diabetes | Type 1 diabetes mellitus |
| Diabetes | Blood glucose increased |
| Diabetes | Blood glucose abnormal |
| Diabetes | Diabetes mellitus management |
| Diabetes | Diabetes mellitus inadequate control |
| Diabetes | Hyperglycaemia |
| Diabetes | Glucose tolerance impaired |
| Diabetes | Glycosylated haemoglobin increased |
| Diabetes | Insulin-requiring type 2 diabetes mellitus |
| Diabetes | Blood glucose decreased |
| Diabetes | Blood glucose |
| Diabetes | Latent autoimmune diabetes in adults |
| Diabetes | Glycosylated haemoglobin |
| Diabetes | Blood glucose fluctuation |
| Diabetes | Hypoglycaemia |
| Diabetes | Glycosylated haemoglobin decreased |
| Diabetes | Insulin resistance |
| Diabetes | Glycosylated haemoglobin abnormal |
| Diabetes | Diabetes prophylaxis |
| Diabetes | Type 3 diabetes mellitus |
| Diabetes | Glycogen storage disease type I |
| Diabetes | Diabetic ketoacidosis |
| Diabetes | Steroid diabetes |
| Diabetes | Diabetic metabolic decompensation |
| Diabetes | Monogenic diabetes |
| Cardiovascular diseases | Diabetic neuropathy |
| Cardiovascular diseases | Cardiac failure |
| Cardiovascular diseases | Cardiac failure chronic |
| Cardiovascular diseases | Cardiac disorder |
| Cardiovascular diseases | Cardiac failure congestive |
| Cardiovascular diseases | Hypertension |
| Cardiovascular diseases | Left ventricular failure |
| Cardiovascular diseases | Heart failure with reduced ejection fraction |
| Cardiovascular diseases | Coronary artery disease |
| Cardiovascular diseases | Dilated cardiomyopathy |
| Cardiovascular diseases | Cardiomyopathy |
| Cardiovascular diseases | Cardiovascular event prophylaxis |
| Cardiovascular diseases | Acute myocardial infarction |
| Cardiovascular diseases | Atrial fibrillation |
| Cardiovascular diseases | Myocardial infarction |
| Cardiovascular diseases | Cardiovascular disorder |
| Cardiovascular diseases | Heart failure with preserved ejection fraction |
| Cardiovascular diseases | Cardiac failure acute |
| Cardiovascular diseases | Myocardial ischaemia |
| Cardiovascular diseases | Ejection fraction decreased |
| Cardiovascular diseases | Cardiac amyloidosis |
| Cardiovascular diseases | Hypertrophic cardiomyopathy |
| Cardiovascular diseases | Acute coronary syndrome |
| Cardiovascular diseases | Cardiac dysfunction |
| Cardiovascular diseases | Angina pectoris |
| Cardiovascular diseases | Arrhythmia |
| Cardiovascular diseases | Ischaemic cardiomyopathy |
| Kidney diseases | Chronic kidney disease |
| Kidney diseases | Renal disorder |
| Kidney diseases | Renal failure |
| Kidney diseases | Renal impairment |
| Kidney diseases | Diabetic nephropathy |
| Kidney diseases | Proteinuria |
| Kidney diseases | Chronic kidney disease-mineral and bone disorder |
| Kidney diseases | IgA nephropathy |
| Kidney diseases | Nephropathy |
| Kidney diseases | Renal disorder prophylaxis |
| Kidney diseases | Nephrotic syndrome |
